# Bendamustine impairs humoral but not cellular immunity to SARS-CoV-2 vaccination in rituximab-treated B-cell lymphoma–affected patients

**DOI:** 10.3389/fimmu.2023.1322594

**Published:** 2023-12-01

**Authors:** Anna Vanni, Lorenzo Salvati, Alessio Mazzoni, Giulia Lamacchia, Manuela Capone, Stefania Francalanci, Seble Tekle Kiros, Lorenzo Cosmi, Benedetta Puccini, Manuel Ciceri, Benedetta Sordi, Gian Maria Rossolini, Francesco Annunziato, Laura Maggi, Francesco Liotta

**Affiliations:** ^1^ Department of Experimental and Clinical Medicine, University of Florence, Florence, Italy; ^2^ Flow Cytometry Diagnostic Center and Immunotherapy, Careggi University Hospital, Florence, Italy; ^3^ Infectious and Tropical Diseases Unit, Careggi University Hospital, Florence, Italy; ^4^ Immunoallergology Unit, Careggi University Hospital, Florence, Italy; ^5^ Hematology Unit, Careggi University Hospital, Florence, Italy; ^6^ Microbiology and Virology Unit, Careggi University Hospital, Florence, Italy; ^7^ Immunology and Cell Therapy Unit, Careggi University Hospital, Florence, Italy

**Keywords:** SARS-CoV-2, mRNA vaccine, B cell lymphoma, anti-CD20mAb, rituximab, bendamustine, humoral response, T cell response

## Abstract

**Background:**

Patients with B-cell lymphoma are a fragile category of subjects, particularly exposed to infections and characterized by an impaired vaccination response due to the disease itself and, even more, to the chemotherapy regimen. For this reason, extensive knowledge of the immune response status of these subjects is of fundamental importance to obtain possible indications for a tailored immunization strategy.

**Methods:**

We enrolled two cohorts of patients with B-cell lymphoma under rituximab treatment or 3–24 months after treatment. In all patients, we evaluated both humoral and cellular immunological memory toward SARS-CoV-2, after standard vaccination and upon one booster dose.

**Results:**

We observed no Spike-specific IgG production in patients (n = 25) under anti-CD20 treatment, whereas patients (n = 16) vaccinated after the completion of chemotherapy showed a higher humoral response. Evaluating SARS-CoV-2–specific T-cell response, we found that patients in both cohorts had developed robust cellular immunity after vaccination. Of the 21 patients (51%) that experienced a breakthrough SARS-CoV-2 infection, only six patients developed severe disease. Interestingly, these six patients had all been treated with rituximab plus bendamustine. Notably, we observed that Spike-specific IgG levels in patients treated with rituximab plus bendamustine were absent or lower compared with those in patients treated with rituximab plus other chemotherapy, whereas Spike-specific T-cell response was not different based on chemotherapy regiment.

**Discussion:**

Our results show that, in patients with B-cell lymphoma under rituximab therapy, anti–SARS-CoV-2 mRNA vaccination induces a weak or absent humoral response but a consistent T-cell response. In addition, chemotherapy regimens with bendamustine further reduce patients’ ability to mount a Spike-specific humoral response even after a long time period from chemotherapy discontinuation. These results provide evidence that different chemotherapeutics display different immunosuppressive properties that could be taken in to account in the choice of the right drug regimen for the right patient. Moreover, they question whether immunocompromised patients, particularly those treated with bendamustine, need interventions to improve vaccine-induced immune response.

## Introduction

COVID-19 pandemic induced the scientific community to deeply investigate the establishment and persistence of immunological memory against SARS-CoV-2 following infection or vaccination. Most studies focused on healthy individuals. In immunocompromised subjects, such as patients with hematologic malignancy receiving immunosuppressive regimens, these aspects are still largely unexplored (1, 2), although secondary immunodeficiency is a major concern for increasing incidence and greatest risk of infection in these patients (3, 4). Rituximab (RTX)–based chemotherapy is widely used in several hematologic malignancies, i.e., non-Hodgkin lymphoma and chronic lymphocytic leukemia (5, 6). RTX is a chimeric monoclonal antibody (mAb) that targets CD20 on B lymphocytes and rapidly induces a prolonged B-cell depletion (5). Usually, circulating B cells are newly detectable 6–9 months after the end of treatment. However, kinetic of B-cell reconstitution could be affected by the concurrent administration of other hematological oncology therapies, such as alkylating agents (bendamustine, chlorambucil, and cyclophosphamide). In these cases, B-cell aplasia can be maintained even longer. Notably, in patients with hematological malignancies, anti–CD20-mAb therapy is administered at a higher dose and generally combined with intensive chemotherapy regimens, in order to enhance the anti-neoplastic effects of RTX but, as a consequence, induce a higher immunosuppression, compared with RTX regimens used in patients with autoimmune diseases (7, 8). Patients receiving these highly immunosuppressive therapies showed a significantly increased risk of infections and a more severe disease course during SARS-CoV-2 infection (9). In addition, because of compromised humoral immune response, these patients displayed impaired vaccine-induced immunity (10–13). Prior studies in patients with autoimmune rheumatic diseases demonstrated that patients treated with RTX have poor humoral immune responses after mRNA or viral vector vaccination (14), but T-cell responses resulted similar between RTX- and non–RTX-treated patients (15). Although T-cell immune response might balance ineffective humoral immunity in patients with B-cell depletion, antibodies are non-redundant and play a critical role for virus clearance. At the moment, there is no consensus on the right time to vaccinate patients with hematological malignancies. A deep analysis with a cellular biology approach will be useful to define how the timing of RTX administration could interfere with vaccine effectiveness, as well as the impact of concomitant immunosuppressive therapies. Hence, we analyzed SARS-CoV-2–specific antibody and CD4^+^ T-cell–mediated immune responses, following standard vaccination cycle and upon booster dose, in patients with hematological malignancy (B-cell lymphoma). Patients were divided into two cohorts: in the first cohort, anti–SARS-CoV-2 vaccination was given under RTX cycle (TP Ongoing), whereas, in the second cohort, after RTX therapy (Post TP). To assess the effect of additional immunosuppressive therapies on vaccine-induced immunity, these two groups were further divided on the basis of the regimen of chemotherapy associated with RTX.

## Materials and methods

### Patients

The study population included 41 patients with B-cell lymphoma recruited at the Careggi University Hospital, Florence, Tuscany, Italy, by the Centre of Research and Innovation of Myeloproliferative Neoplasms. The first cohort (TP Ongoing, n = 25) consisted of patients with B-cell lymphoma who received the first two doses of anti–SARS-CoV-2 vaccine while they were under RTX therapy, whereas the second cohort (Post TP, n = 16) included patients with B-cell lymphoma who completed RTX therapy between 3 and 12 months before anti–SARS-CoV-2 vaccination. Patients were stratified on the basis of the concomitant chemotherapy regimen received, particularly considering the administration of bendamustine. Demographic and clinical features of enrolled patients are summarized in **Table 1**. Additional laboratory data on hemoglobin level, leucocyte, lymphocyte, and platelet counts in recruited patients at T0 are reported in **Supplementary Table 1**. Patients received the first mRNA-1273 vaccine dose in March 2021, followed by a second mRNA-1273 vaccine dose 28 days later. Patients received a third vaccine dose between December 2021 and January 2022. A blood draw was performed at basal time (time 0, before the first vaccine dose), 28 days after (T1, before the second dose), 60 days after (T2), and 1 month after the third vaccine dose (T3). None of the study participants had prior PCR-confirmed SARS-CoV-2 infection, IgG antibodies against nucleoprotein and T-cell response against nucleoprotein and membrane protein (**Supplementary Figure 1**).

**Table 1:** Main demographic and clinical data of patients with B-cell lymphoma receiving anti–SARS-CoV-2 mRNA vaccination under rituximab (n = 25) or post-rituximab therapy (n = 16).

	ID	Age at vaccination (years)	Gender	Disease	Treatment protocol	Time between rituximab therapy and first vaccine dose (days)	Time from latest bendamustine administration to first vaccine dose (days)	SARS-CoV-2 infection after vaccination; WHO severity
**Ongoing rituximab therapy (TP-Ongoing)**	ON1B	80	M	FL	R-BENDA; RTX	NA	383	Yes; 4
	ON2	43	M	GZL	DA-EPOCH-R	NA	NA	No
	ON3B	70	F	LL	R-BENDA	10	10	No
	ON4	79	F	PBCL	RTX	NA	NA	Yes; 1
	ON5	73	M	FL	R-CHOP; RTX	12	NA	No
	ON6B	75	M	FL	R-BENDA; RTX	NA	309	Yes; 1
	ON7B	75	F	FL	BENDA; R-CVP; RTX	NA	521	Yes; 2
	ON8	47	F	DLBCL	R-CHOP	NA	NA	Yes; 2
	ON9B	75	F	FL	R-BENDA; RTX	NA	478	No
	ON10B	72	M	FL	R-BENDA; RTX	NA	712	No
	ON11B	53	F	FL	R-BENDA; RTX	NA	444	Yes; 2
	ON12B	56	F	FL	R-BENDA; RTX	NA	505	Yes; 1
	ON13	63	F	DLBCL	R-COMP; MTX; R-DHAOx	NA	NA	No
	ON14	52	M	DLBCL	R-CHOP; R-DHAOx; RTX	NA	NA	Yes; 1
	ON15B	65	F	FL	R-CHOP; R-BENDA; RTX	NA	675	Yes; 3
	ON16B	71	F	FL	R-BENDA; RTX	NA	504	No
	ON17B	53	F	FL	R-BENDA; RTX	NA	496	Yes; 3
	ON18B	52	M	DLBCL	R-CHOP; R-DHAOx; PIX; BENDA	21	NA	Yes; 4
	ON19B	61	M	FL	R-BENDA; RTX	NA	530	Yes; 4
	ON20	46	M	DLBCL	R-CHOP	34	NA	No
	ON21	54	M	DLBCL	R-CHOP*	33**	NA	Yes; 1
	ON22	67	M	DLBCL	R-CHOP*	24**	NA	Yes; 1
	ON23	74	M	DLBCL	R-COMP*	14**	NA	Yes; 1
	ON24	59	F	DLBCL	R-CHOP*	10**	NA	Yes; 1
	ON25	37	F	PMBCL	R-CHOP*	7**	NA	Yes; 2
	Mean	62.1	–	–	–	–	–	–
	SD	12.3	–	–	–	–	–	–
	Median	63	–	–	–	–	–	–
	IQR	20	–	–	–	–	–	–
	M/F ratio	–	0.9	–	–	–	–	–
**Post rituximab therapy (Post-TP)**	POST1B	56	F	SMZL	R-BENDA; RTX	163	285	Yes; 1
	POST2	63	M	FL	RTX	141	NA	Yes; 1
	POST3B	73	M	LPL	R-BENDA	409	409	No
	POST4	70	M	DLBCL	R-CHOP; RTX	98	NA	No
	POST5B	69	M	MCL	R-BAC	77	NA	No
	POST6	73	M	DLBCL	R-CHOP	284	NA	No
	POST7B	74	M	MCL	R-BENDA	311	311	No
	POST8	68	M	DLBCL	R-COMP	223	NA	No
	POST9B	72	M	MCL	R-FND; RTX; R-BENDA	120	120	No
	POST10	64	M	MCL	R-CHOP; R-DHAOx	322	NA	No
	POST11B	80	M	MCL	R-BENDA	588	588	No
	POST12B	46	F	NMZL	R-BENDA	71	71	No
	POST13	68	F	NMZL	RTX	185	NA	No
	POST14B	69	F	MCL	RTX; R-BENDA; RTX	125	619	Yes; 4
	POST15	57	M	BL	R-COMP; MTX	186	NA	No
	POST16	78	M	DLBCL	R-COMP	100	NA	Yes; 1
	Mean	67.5	–	–	–	212.7	–	–
	SD	8.7	–	–	–	140.5	–	–
	Median	69	–	–	–	174	–	–
	IQR	9.3	–	–	–	175.8	–	–
	M/F ratio	–	3	–	–	–	–	–

BCL, B-cell lymphoma; BL, Burkitt lymphoma; DA-EPOCH-R, dose-adjusted etoposide, prednisone, vincristine, cyclophosphamide, doxorubicin, and rituximab; DLBCL, diffuse large B-cell lymphoma; F, female; FL, follicular lymphoma; GZL, gray zone lymphoma; LL, lymphocytic lymphoma; LPL, lymphoplasmacytic lymphoma; M, male; MCL, mantle cell lymphoma; MTX, methotrexate; NA, not applicable; NMZL, nodal marginal zone lymphoma; PBCL, peripheral B-cell lymphoma; PIX, pixantrone; PMBCL, primary mediastinal large B-cell lymphoma; R-BAC, rituximab, bendamustine, cytarabine; R-BENDA, rituximab and bendamustine; R-CHOP, rituximab, cyclophosphamide, doxorubicin, prednisone, and vincristine; R-COMP, rituximab, cyclophosphamide, non-pegylated liposomal doxorubicin, prednisone, and vincristine; R-CVP, rituximab, cyclophosphamide, vincristine, and prednisolone; R-DHAOx, rituximab, dexamethasone, cytarabine, and oxaliplatin; R-FND, rituximab, fludarabine, mitoxantrone, and dexamethasone; SMZL, splenic marginal zone lymphoma.

*Treatment protocol followed after first vaccine dose administration.

**Time from first vaccine dose administration to start of rituximab therapy.

Peripheral blood mononuclear cells (PBMNCs) were obtained following density gradient centrifugation of blood samples using Lymphoprep (Axis Shield Poc As™) and were frozen in Fetal calf serum (FCS) plus 10% Dimethyl Sulfoxide (DMSO) to be stored in liquid nitrogen. For each subject, longitudinal samples were defrosted and analyzed together for T-cell evaluation. Sera were frozen and stored for Ig level evaluation.

### Characteristics of study population

A total of 41 patients with B-cell lymphoma were assessed for serum antibody analysis and T-cell response evaluation (**Table 1**). The majority of them (n = 25) was under RTX therapy, whereas 16 patients had already completed RTX treatment. For the latter group, the median time from last therapy cycle and first vaccine dose was 174 days. The median age (range) for TP-Ongoing patients was 63 (37–80) years with a male/female ratio of 0.9, whereas, for the Post-TP group, the median age was 69 (46–80) with a male/female ratio of 3. The most common conditions were follicular lymphoma (31.7%) and diffuse large B-cell lymphoma (31.7%), followed by mantle cell lymphoma (14.6%) and nodal marginal zone lymphoma (4.9%). In the TP-Ongoing group, only one patient received RTX therapy alone, whereas the other 24 patients received RTX in combination with intensive chemotherapy regimens. In particular, 13 patients were under RTX treatment and a regimen that included bendamustine, whereas 11 patients were treated with RTX plus other chemotherapy, including R-CHOP (RTX, cyclophosphamide, doxorubicin, prednisone, and vincristine), R-COMP (RTX, cyclophosphamide, non-pegylated liposomal doxorubicin, prednisone, and vincristine), and others (**Table 1**). Regarding the Post-TP cohort, two patients were treated with RTX alone, eight received RTX in combination with a regimen that included bendamustine, and six were treated with RTX plus other chemotherapy (**Table 1**).

### Evaluation of SARS-CoV-2 Spike–reactive T cells

For T-cell stimulation *in vitro*, 1.5 million PBMNCs were cultured in complete RPMI plus 5% human AB serum in 96-well flat bottom plates in the presence of medium alone (background, negative control) or of a pool of Spike SARS-CoV-2 peptide pools (Prot_S1, Prot_S^+^, and Prot_S to achieve a complete sequence coverage of the Spike protein) at 0.6 µM/peptide, according to the manufacturer’s instructions (Miltenyi Biotec). T-cell response against nucleoprotein (N) peptide pool (Miltenyi Biotec) was assessed as well. After 2 h of incubation at 37°C, 5% CO_2_, brefeldin A (5 µg/mL) was added, followed by additional 4h incubation. Finally, cells were fixed and stained using fluorochrome-conjugated antibodies listed in **Supplementary Table 2**. Samples were acquired on a BD LSR II flow cytometer (BD Biosciences) using the gating strategy showed in **Supplementary Figure 2**. Flow cytometry experiments were performed using published guidelines (16).

### Evaluation of SARS-CoV-2–specific IgG

Evaluation of SARS-CoV-2 Spike–specific IgG (Diasorin), nucleoprotein-specific IgG (Abbott), and Receptor Binding Domain (RBD)-specific IgG (Abbott) were performed following the manufacturer’s instructions. The antibody reactivity of each specimen was expressed by the ratio between optical density and cutoff value (index) or as binding antibody unit (BAU)/ml.

### Evaluation of circulating CD19^+^ B cells

Before T-cell evaluation, all defrosted PBMNCs were evaluated by flow cytometry for the presence of circulating CD19^+^ B cells. In detail, PBMNCs were stained using fluorochrome-conjugated antibodies CD3-Pacific Blue and CD19-APC-Cy7 (BD Biosciences), in order to assess the percentage of lymphocyte expressing CD19. Flow cytometry experiments were performed using the published guidelines (16).

### Statistics

Unpaired Mann–Whitney test was used to compare TP-Ongoing versus Post-TP cohorts and to compare patients treated with bendamustine versus those receiving regimens without bendamustine. Wilcoxon signed-rank test was used to compare different time points of analysis in each study group. In all cases, *p* values ≤ 0.05 were deemed as significant.

### Study approval

The procedures followed in the study were approved by the Careggi University Hospital Ethical Committee (CEAVC 23847_BIO). Written informed consent was obtained from recruited patients.

## Results

### Higher Spike-specific antibodies after COVID-19 vaccination in Post-TP patients compared with that in TP-Ongoing patients

**Figure 1:**
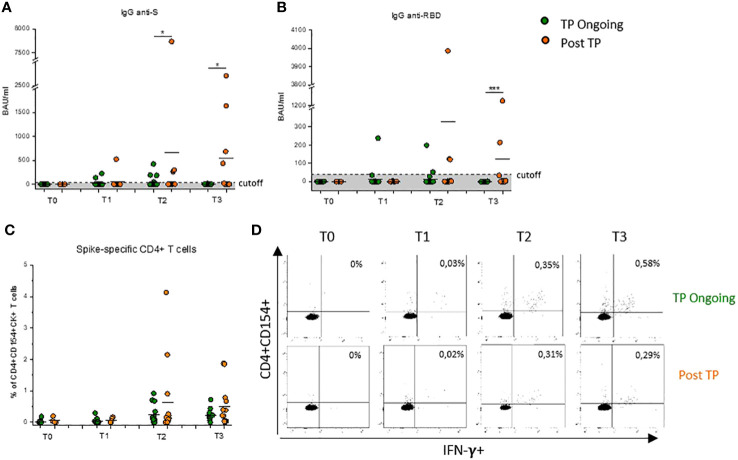
Evaluation of Spike-specific antibodies and CD4^+^ T cells after COVID-19 vaccination in Ongoing-TP and Post-TP patients. Evaluation of IgG specific for Spike protein **(A)** or RBD **(B)** in TP-Ongoing patients (green dots) at T0 (n = 24), T1 (n = 23), T2 (n = 23), and T3 (n = 10) and in Post-TP (orange dots) cohort at T0 (n = 3), T1 (n = 9), T2 (n = 13), and T3 (n = 12). **(C)** Frequency of CD4^+^ T cells reactive to SARS-CoV-2, defined by expression of CD154 and at least one cytokine among IFN-γ, IL-2, and TNF-α (CD4^+^CD154^+^CK^+^) in TP-Ongoing patients (green dots) at T0 (n = 14), T1 (n = 15), T2 (n = 15), and T3(n = 10) and in Post-TP (orange dots) cohort at T0 (n = 3), T1 (n = 7), T2 (n = 13), and T3 (n = 12). **(D)** Representative flow cytometric plots of Spike-specific CD154^+^CD4^+^IFN-γ^+^ T cells, at each time point, in one selected TP-Ongoing individual (upper panels) and Post-TP individual (lower panels). Black lines in **(A–C)** represent mean values, and gray area delimited by a dotted line in **(A)** and **(B)** represents the cutoff value. The evaluation time points are T0 (before vaccination), T1 (before the second dose), T2 (1 month after the second dose), and T3 (1 month after the third dose). *p < 0.05 and ***p < 0.001 calculated with Mann–Whitney U-test.

Sera of both TP-Ongoing and Post-TP patients were analyzed for IgG anti-Spike and IgG anti-RBD. At baseline (T0), no antibodies against Spike or RBD were detected in all recruited patients (**Figures 1A, B**). Because of the complete deletion of CD20^+^ B cells, SARS-CoV-2–specific IgGs were undetectable in almost all patients receiving vaccination during RTX treatment (TP Ongoing) (**Figures 1A, B**). Interestingly, among the group of TP-Ongoing patients, two subjects at T1 (after the first dose) and three subjects at T2 (1 month after the second dose) showed detectable low levels of anti-RBD and anti-Spike IgGs (**Figures 1A, B**). Notably, two of these patients (ON18 and ON22) started RTX therapy shortly after the first vaccine dose (24 and 21 days, respectively) but completed the vaccination cycle while under RTX treatment (**Table 1**). Indeed, these two patients displayed detectable CD19^+^ cells at T0 (8.1% and 4.2% of CD19^+^ B lymphocyte, respectively), whereas no CD19^+^ cells were detected at T1, T2, and T3 (data not shown).

Conversely, patients in the Post-TP cohort, having completed the standard vaccination cycle at least 3 months after discontinuation of RTX/chemotherapy, showed appreciable humoral Spike–specific response at T2 and T3 compared with TP-Ongoing patients, reaching a statistical significance for anti-Spike IgG both at T2 and T3 and for anti-RBD IgG only at T3 (**Figures 1A, B**). It is important to underline that IgG levels observed in RTX-treated patients remain lower compared with those observed in healthy subjects (17). Notably, no correlation was found between time from last RTX administration and anti-Spike or anti-RBD IgG levels observed at both T2 and T3 time points (**Supplementary Figure 3**).

### Spike-specific CD4^+^ T-cell response is present and intense in both Ongoing-TP and Post-TP patients after COVID-19 mRNA vaccination

RTX therapy induced a rapid B-cell depletion with no effect on T-cell numbers. Nevertheless, the majority of patients with B-cell lymphoma included in this study received intensive chemotherapy regimens in combination with RTX, to influence both quantitatively and functionally T cells. For this reason, the ability of CD4^+^ T cells to be re-activated *in vitro* upon stimulation with a pool of peptides covering the entire sequence of the Wuhan-strain (wild type) Spike was investigated. Both TP-Ongoing and Post-TP patients showed a robust cell–mediated response with high level of Spike-specific CD4^+^ T cells at T1, T2, and T3, with no difference between the two cohorts (**Figures 1C, D**). Notably, percentages of Spike-specific CD4^+^ T cells in RTX-treated patient were comparable to those observed in healthy subjects (**Supplementary Figure 4**) (17).

### RTX + bendamustine–treated patients with B-cell lymphoma showed more severe COVID-19 compared with RTX + other chemotherapy–treated patients

**Figure 2:**
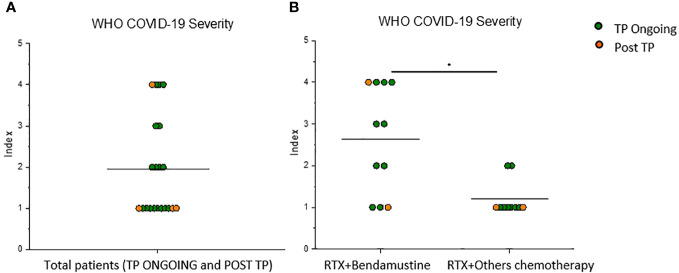
RTX + bendamustine–treated patients showed a more severe disease course during SARS-CoV-2 infection. **(A)** WHO COVID-19 severity index of 21 lymphoma B-cell patients (TP Ongoing, n = 17, green dots) (Post TP, n = 4, orange dots) infected with SARS-CoV-2 after vaccination and **(B)** WHO COVID-19 severity index of 11 RTX + bendamustine–treated patients and 10 RTX + other chemotherapy–treated patients. Mean values are represented as black lines. *P < 0.05 calculated with Mann–Whitney U-test.

Because of lower Spike-specific humoral response in the analyzed RTX-treated cohort compared with reported data on healthy subjects (17), we prospectively assessed the incidence and severity of breakthrough infections in the study population in 1-year follow-up after vaccination. Among all recruited patients, both Ongoing-TP and Post-TP cohorts, breakthrough SARS-CoV-2 infections were reported in 21 subjects (3–12 months after T3) with different severity; in particular, five of the 21 patients showed a more severe diseases course (**Figure 2A**), according to WHO COVID-19 severity index. In the TP-Ongoing cohort, 68% of patients developed COVID-19, which was most commonly mild (8 of 17) or moderate (4 of 17). Only three and two of the 17 patients developed critical disease and severe disease, respectively. On the contrary, in the Post-TP cohort, 25% of patients developed COVID-19, which, in the majority of patients (three of four), was mild. Notably, all the five patients who showed a higher WHO COVID-19 severity index (score of 3 or 4) were RTX + bendamustine–treated patients. Therefore, dividing all infected patients on the basis of bendamustine therapy, we interestingly observed that RTX + bendamustine–treated patients showed a significantly higher WHO COVID-19 severity index (mean = 2.4) compared with RTX + other chemotherapy–treated patients (mean = 1.2) (**Figure 2B**). No difference in the number of breakthrough infections was observed between the two analyzed cohort (data not shown).

### Chemotherapy regimen associated with RTX influences the humoral vaccine–induced immune response

**Figure 3:**
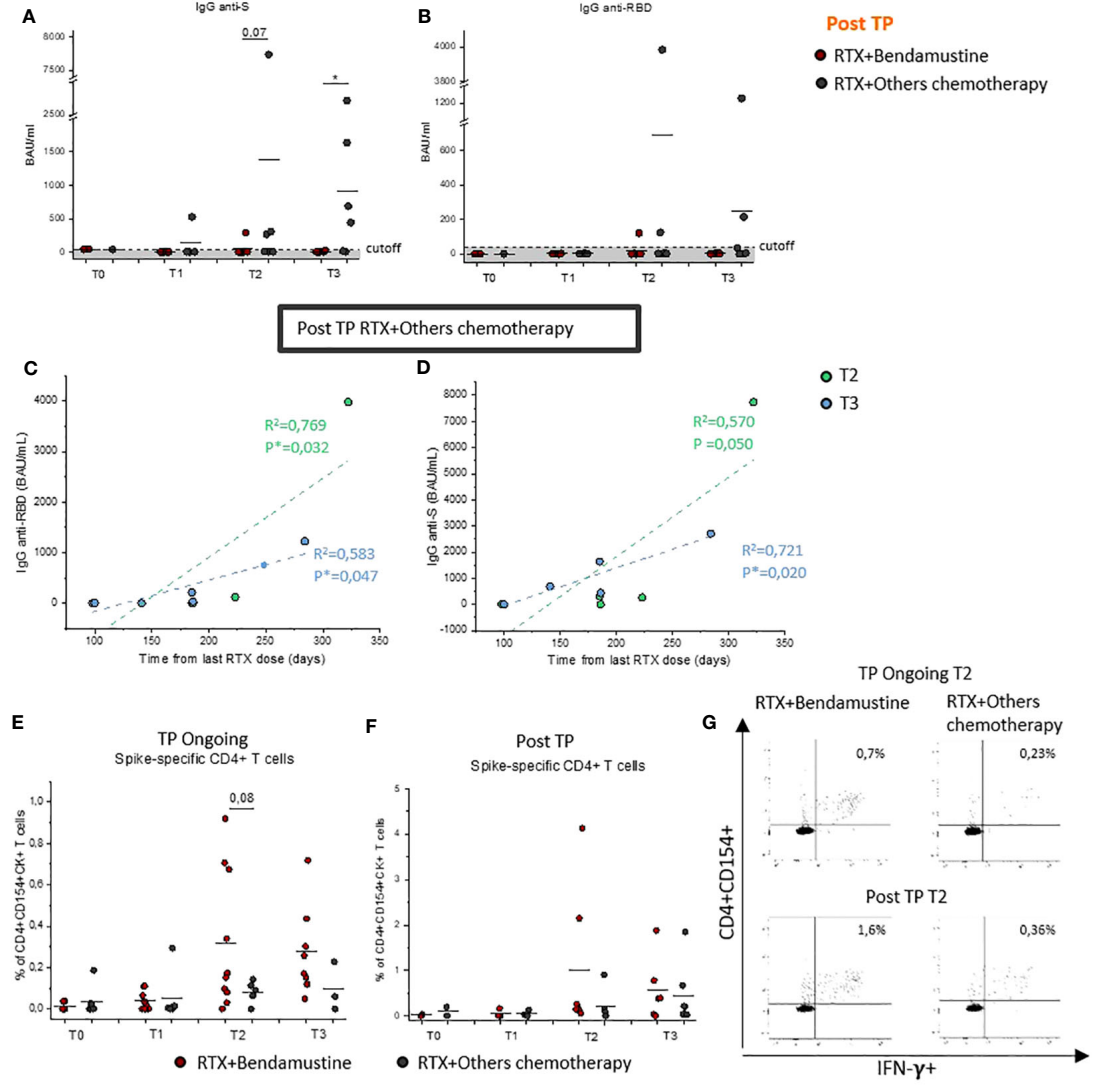
Evaluation of Spike-specific antibodies and CD4^+^ T cells after COVID-19 vaccination in bendamustine- and no-bendamustine–treated patients. Evaluation of IgG specific for Spike protein **(A)** or RBD **(B)** in Post-TP RTX + bendamustine–treated patients (red dots) at T0 (n = 2), T1 (n = 5), T2 (n = 6), and T3 (n = 6) and RTX + other chemotherapy–treated patients (gray dots) at T0 (n = 1), T1 (n = 4), T2 (n = 7), and T3 (n = 6). Correlation between IgG anti-S **(C)** or IgG anti-RBD **(D)** with time from last rituximab dose on total Post-TP RTX + other chemotherapy–treated group at T2 (n = 7; light green dots) or T3 (n = 6; light blue dots). Pearson’s correlation coefficients were used to calculate the correlations; statistical significance (*p < 0.05) is reported on each graph. **(E)** Frequency of CD4^+^ T cells reactive to SARS-CoV-2 in TP-Ongoing RTX + bendamustine–treated patients (red dots) at T0 (n = 7), T1 (n = 9), T2 (n = 10), and T3 (n = 8) and RTX + other chemotherapy–treated patients (gray dots) at T0 (n = ), T1 (n = 6), T2 (n = 5), and T3 (n = 3). **(F)** Frequency of CD4^+^ T cells reactive to SARS-CoV-2 in Post-TP RTX + bendamustine–treated patients (red dots) at T0 (n = 2), T1 (n = 3), T2 (n = 6), and T3 (n = 5) and RTX + other chemotherapy–treated patients (gray dots) at T0 (n = 1), T1 (n = 4), T2 (n = 7), and T3(n = 6). **(G)** Representative flow cytometric plots of Spike-specific CD154^+^CD4^+^IFN-γ^+^ T cells, at T2, in two selected TP-Ongoing individuals (upper panels) and in two selected Post-TP individuals (upper panels) RTX + bendamustine–treated or RTX + other chemotherapy–treated, left and right panels, respectively. Black lines in **(A, B, E, F)** represent mean values, and gray area delimited by a dotted line in **(A)** and **(B)** represents the cutoff value. *p < 0.05 calculated with Mann–Whitney U-test.

Given the increased risk to develop a more severe diseases course after SARS-CoV-2 infection in RTX + bendamustine–treated patients, cohorts were subsequently divided on the basis of the type of chemotherapy regimen associated with RTX. We observed that IgG levels were also influenced by the type of chemotherapy received; in particular, Post-TP patients that had been previously treated with RTX + bendamustine showed a lower or absent level of SARS-CoV-2–specific IgGs compared with Post-TP patients treated with RTX + other chemotherapy (**Figures 3A, B**). Interestingly, considering only patients treated with RTX + other chemotherapy, a statistically significant positive correlation between anti-Spike and anti-RBD IgG levels and time from the last RTX dose (**Figures 3C, D**) was found both at T2 and T3.

T-cell–mediated response was also evaluated considering the presence of bendamustine in the chemotherapy regimen. Our data highlight that T-cell–mediated response in both TP-Ongoing and Post-TP patients was not significantly influenced by chemotherapy regimen compared with the humoral response, showing that it, if anything, had an opposite trend. In fact, RTX + bendamustine–treated patients showed comparable frequency of CD4^+^ Spike-specific T cells compared with patients whose regimens did not include bendamustine (**Figures 3E–G**).

## Discussion

The effectiveness of SARS-CoV-2 mRNA vaccines has been demonstrated by several studies performed throughout 2021 on different cohorts, mainly including healthy individuals (17, 18). SARS-CoV-2 infection can present asymptomatically or mildly but may also cause severe immune dysregulation, leading to hospitalization and poor outcome (19). Patients with B-cell lymphoma have an increased risk of severe COVID-19 with fatal outcome. In fact, hematological malignancies represent an independent risk factor for COVID-19 mortality (20–22). Effective vaccination against SARS-CoV-2 would contribute to the protection of these patients against severe COVID-19. There are only a few studies reporting data on the vaccine-induced immune protection in this specific population (23–25). These studies mainly focus on the effects of RTX therapy on humoral vaccine–induced response, with low or absent consideration of concomitant high immunosuppressive chemotherapy to which patients with B-cell lymphoma are exposed (25). It is therefore important to investigate whether these patients benefit from current vaccines and what additional strategy should be eventually adopted to guarantee vaccine effectiveness. To this end, in the current study, the humoral and cellular responses to two and three doses of COVID-19 mRNA vaccine in patients with B-cell lymphoma were evaluated, focusing on RTX therapy and investigating the effect of additional immunosuppressive chemotherapy on vaccine-induced response.

Our results showed, consistent with previous reports (26–28), that hematological patients who had been previously exposed to anti-CD20 mAb therapy had a poor/absent humoral response to anti–SARS-CoV-2 mRNA vaccination, very likely due to therapy-related B-cell deficiency. Interestingly, patients undergoing vaccination at least 3 months after the interruption of RTX therapy showed higher antibodies response. This result could be predictable due to the re-establishment of the B-cell compartment in RTX-treated patients 6–12 months after therapy (5). As a result, B cells could therefore respond to vaccine stimuli and start to produce antibodies. Nevertheless, in our whole cohort of Post-TP patients, no correlation was found between time from last RTX administration and antibodies titer, which was instead observed in other cohorts of patients (25, 29). Notably, this correlation is observed when we only consider patients whose chemotherapeutic regimen does not include bendamustine in association with RTX. Therefore, not only anti-CD20 mAb but also the type of chemotherapy received could influence the re-establishment of B-cell compartment and therefore humoral response to vaccines. Among administered chemotherapies, because of its efficacy and favorable toxicity profile, bendamustine has become one of the preferred therapeutic choices for the treatment of indolent B-cell lymphoma together with R-CHOP treatment (30, 31). Nevertheless, myelosuppression including lymphopenia occurs more frequently in patients receiving combinations of bendamustine and RTX compared with those in patient treated with CHOP and RTX (32–34). In agreement with these data, in our restricted cohort of Post-TP patients, we observed a lower or absent humoral response to anti–SARS-CoV-2 mRNA vaccination and no correlation between last RTX dose and antibodies titers in only patient treated with RTX + bendamustine compared with those in patient treated with RTX + other chemotherapy regimens. These data show evidence of a stronger and prolonged immunosuppression in B-cell compartment in patients receiving bendamustine in addition to anti-CD20 therapy.

Not only humoral response is essential for neutralization of virus entrance in the host cells, for virus clearance, and for preventing development of severe disease, but also T cells play a fundamental role in the prevention of more severe disease course during SARS-CoV-2 infection. In both enrolled cohorts of hematological patients, a robust Spike-specific CD4^+^ T-cell response to anti–SARS-CoV-2 mRNA vaccination is established and maintained, already after two vaccine doses. The observed cell-mediated response was comparable and, in some cases, even higher to previously studied healthy subjects (17). On a restricted numbers of TP-Ongoing patients at T2 and T3 (three and four patients, respectively), we had the opportunity to test CD4^+^ T-cell response against omicron variant (data not shown). Interestingly, the global CD4^+^ T-cell response of these patients is retained also against Omicron, in accordance with our previous study performed on healthy individuals (35).

Unexpectedly, T-cell response seemed not to be negatively influenced by RTX or chemotherapy regimens associated with RTX. Indeed, patients treated with RTX + bendamustine showed a comparable percentage of Spike-specific CD4^+^ T cells, compared with patients treated with other chemotherapy. This observation might indicate that cell-mediated response compensates, at least in part, the lack of the humoral counterpart. Nevertheless, Spike-specific T-cell response was evaluated in terms of frequency of cells and not of absolute cell number that could be lower in these immunocompromised patients given that lymphopenia occurs relatively frequently after therapy with bendamustine (36). This could be one of the reasons explaining why RTX + bendamustine–treated patients showed a higher propensity to develop more severe COVID-19, even if their percentage of Spike-specific CD4^+^ T-cell response was comparable to RTX + other chemotherapy–treated patients. The higher severity of SARS-CoV-2 breakthrough infection observed in RTX + bendamustine–treated cohort is in accordance with the highly immunosuppressive status caused by the association of RTX plus bendamustine, given that for other pathogens it was already reported that bendamustine-treated patients show increased risk of infection (36).

These results question whether immunocompromised patients, such as patients with B-cell lymphoma treated with RTX and associated chemotherapy regimens, need intervention to improve SARS-CoV-2 mRNA vaccine response or response to other common vaccines. These interventions could comprise improvement of the vaccination schedule by modifying time length between the second and third injection, by additional vaccine doses, or by modulation/interruption of concomitant immunosuppressive therapy, such as bendamustine. Nevertheless, whether this optimization might result in an improved level of protection remains uncertain, and further studies are essential to better define this prospective.

## Conclusions

Our results show that, in hemato-oncological patients vaccinated while under RTX therapy, anti–SARS-CoV-2 mRNA vaccination induces a weak or absent humoral response, but a consistent T-cell–mediated response is present, which seems to counterbalance the lack of the humoral response. In addition, chemotherapy regimens comprising bendamustine further reduced patients’ ability to mount a Spike-specific humoral response even after a long time period from therapy discontinuation. These results question whether immunocompromised patients, particularly those treated with bendamustine, need interventions to improve vaccine-induced immune response such as additional vaccine doses over time or modulation of vaccination schedule in respect to timing of immunosuppressive therapy. Moreover, these results offer a biological base that may be useful for the clinicians, in order to better stratify the infection risk of their patients, improving the accuracy in the choice of the drugs for a tailored chemotherapeutic regimen.

## Data availability statement

The original contributions presented in the study are included in the article/**Supplementary Material**. Further inquiries can be directed to the corresponding author.

## Ethics statement

The studies involving humans were approved by Comitato Etico Regionale Area Vasta Centro Toscana, Italia. The studies were conducted in accordance with the local legislation and institutional requirements. The participants provided their written informed consent to participate in this study.

## Author contributions

AV: Data curation, Formal Analysis, Investigation, Methodology, Validation, Writing – original draft. LS: Data curation, Formal Analysis, Investigation, Validation, Writing – original draft. AM: Data curation, Formal Analysis, Investigation, Methodology, Validation, Writing – original draft. GL: Data curation, Investigation, Methodology, Writing – review & editing. MCa: Data curation, Formal Analysis, Investigation, Methodology, Writing – review & editing. SF: Data curation, Formal Analysis, Investigation, Methodology, Writing – review & editing. SK: Data curation, Formal Analysis, Investigation, Methodology, Writing – review & editing. LC: Data curation, Supervision, Validation, Writing – review & editing. BP: Data curation, Supervision, Validation, Writing – review & editing. MCi: Methodology, Writing – review & editing. BS: Methodology, Writing – review & editing. GR: Data curation, Supervision, Validation, Writing – review & editing. FA: Conceptualization, Funding acquisition, Project administration, Supervision, Validation, Writing – original draft. LM: Data curation, Formal Analysis, Funding acquisition, Investigation, Validation, Writing – original draft. FL: Conceptualization, Project administration, Supervision, Validation, Writing – original draft.
