# Supplementary material for: Bendamustine impairs humoral but not cellular immunity to SARS-CoV-2 vaccination in rituximab-treated B-cell lymphoma–affected patients
Source: Front Immunol. 2023 Dec 1;14:1322594. doi: 10.3389/fimmu.2023.1322594 (PMC10722279; doi:10.3389/fimmu.2023.1322594)
Supplement: Supplementary file 1 [file DataSheet_1.docx]

**SUPPLEMENTARY TABLES**

**Supplementary Table 1.** Hemoglobin level, leucocyte, lymphocyte and platelet counts in patients with B-cell lymphoma receiving anti-SARS-CoV-2 mRNA vaccination under rituximab (n=25) or post-rituximab therapy (n=16).

**Supplemental Table 2.** List of all fluorochrome-conjugated antibodies used for flow cytometric analysis of Spike-specific T cells.

**SUPPLEMENTARY FIGURES**

**Supplemental Figure 1.** Humoral and cellular patients’ immune response against Nucleoprotein and Membrane protein.

**Supplemental Figure 2**. Gating strategy for the identification of SARS-CoV-2-specific T cells.

**Supplemental Figure 3**. Correlation between IgG levels and the time from last rituximab dose on post therapy group.

**Supplemental Figure 4.** Evaluation of Spike specific CD4+ T cells after COVID-19 vaccination in Ongoing TP, Post TP patients and Healthy subjects,

|  | **ID** | **Hemoglobin level**  **(g/dL)** | **Leucocyte count**  **(cells/µL)** | **Lymphocyte count**  **(cells/µL)** | **Platelet count**  **(cells/µL)** |
| --- | --- | --- | --- | --- | --- |
| **Ongoing rituximab therapy (TP Ongoing)** | ON1B | 14,5 | 4,5 | 1,9 | 168 |
|  | ON2 | 11,9 | 8,61 | 0,87 | 406 |
|  | ON3B | 12,9 | 4,79 | 0,68 | 170 |
|  | ON4 | 13,1 | 4 | 1,91 | 164 |
|  | ON5 | 13,4 | 2,95 | 0,58 | 197 |
|  | ON6B | 14 | 3,21 | 0,58 | 185 |
|  | ON7B | 11,1 | 2,6 | 0,89 | 174 |
|  | ON8 | 11,2 | 3,06 | 0,77 | 254 |
|  | ON9B | 12,6 | 6,24 | 1,45 | 214 |
|  | ON10B | 11,8 | 1,83 | na | 179 |
|  | ON11B | 13,2 | 6,3 | 1 | 300 |
|  | ON12B | 14,3 | 5,3 | na | 174 |
|  | ON13 | 10,7 | 3,64 | na | 323 |
|  | ON14 | 16,4 | 6,72 | 2,04 | 278 |
|  | ON15B | 12,4 | 4,54 | 0,99 | 236 |
|  | ON16B | 13 | 11,6 | 1,43 | 266 |
|  | ON17B | 13,1 | 2,6 | 0,52 | 247 |
|  | ON18B | 9,5 | 4,71 | 0,3 | 66 |
|  | ON19B | 15,5 | 5,9 | na | 150 |
|  | ON20 | 15,5 | 4,89 | 1,59 | 156 |
|  | ON21 | 10,5 | 3,48 | 1,14 | 325 |
|  | ON22 | 12,8 | 6,7 | 0,66 | 267 |
|  | ON23 | 12,1 | 3,66 | 0,22 | 287 |
|  | ON24 | 9,2 | 2,13 | 0,86 | 171 |
|  | ON25 | 10,1 | 6,15 | na | 325 |
|  | Mean | 12,6 | 4,8 | 1,0 | 227,3 |
|  | SD | 1,9 | 2,2 | 0,5 | 75,8 |
|  | Median | 12,8 | 4,5 | 0,9 | 214,0 |
|  | IQR | 2,2 | 2,9 | 0,8 | 107,0 |
| **Post rituximab therapy (Post TP)** | POST1B | 13,8 | 6,02 | na | 259 |
|  | POST2 | 15,7 | 5,17 | 1,19 | 230 |
|  | POST3B | 14,9 | 7,29 | 0,73 | 287 |
|  | POST4 | 13,7 | 5,15 | 1,34 | 298 |
|  | POST5B | 7,9 | 11,5 | 2,97 | 295 |
|  | POST6 | 14 | 12,9 | na | 218 |
|  | POST7B | 14 | 5,05 | 1,59 | 142 |
|  | POST8 | 15,1 | 9,4 | na | 229 |
|  | POST9B | 15 | 4,87 | 1,27 | 238 |
|  | POST10 | 10,2 | 3,6 | 0,72 | 95 |
|  | POST11B | 16,7 | 2,74 | 0,36 | 153 |
|  | POST12B | 13 | 2,94 | 0,68 | 166 |
|  | POST13 | 13,3 | 3,1 | 0,7 | 110 |
|  | POST14B | 12,5 | 2,2 | 0,58 | 201 |
|  | POST15 | 13,3 | 2,89 | 0,96 | 124 |
|  | POST16 | 11,8 | 4,81 | 1,4 | 244 |
|  | Mean | 13,4 | 5,6 | 1,1 | 205,6 |
|  | SD | 2,1 | 3,2 | 0,7 | 66,5 |
|  | Median | 13,8 | 5,0 | 1,0 | 223,5 |
|  | IQR | 3,9 | 3,4 | 0,7 | 100,3 |
|  | *p*-value* | 0.15272 | 0.63836 | 0 .9124 | 0.34722 |

**Supplementary Table 1.** Hemoglobin level, leucocyte, lymphocyte and platelet counts in patients with B-cell lymphoma receiving anti-SARS-CoV-2 mRNA vaccination under rituximab (n=25) or post-rituximab therapy (n=16).

* p-values obtained with Mann-Whitney U test (Wilcoxon rank-sum test) comparing the two groups. The p-

values indicate the statistical significance of the difference between the two groups for each variable.

| **Antigen** | **Fluorochrome** | **Clone** | **Company** |
| --- | --- | --- | --- |
| TNF-α | FITC | 6401.1111 B | BDBioscience |
| CD154 | PE | TRAP1 | BDBioscience |
| CD3 | PerCP | SK7 | BDBioscience |
| CD4 | PECy-7 | SK3 | Invitrogen |
| CD8 | SB600 | SK1 | eBioscience™ |
| IL-2 | APC | MQ1-17H12 | BDBioscience |
| IFN-γ | Pacific Blue | B27 | BioLegend |
| L/D | Fixable Viability Stain 780 |  | BDBioscience |

**Supplemental Table 2.** List of all fluorochrome-conjugated antibodies used for flow cytometric analysis of Spike-specific T cells.


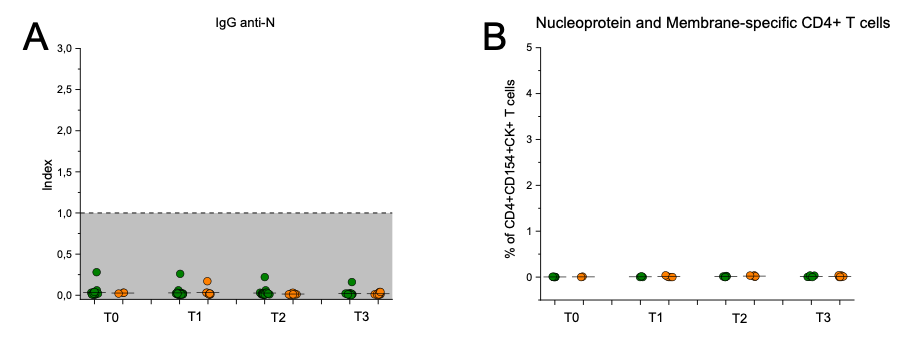
**Supplemental Figure 1.** Humoral and cellular patients’ immune response against Nucleoprotein and Membrane protein.

Evaluation of IgG specific for Nucleoprotein (A) in TP Ongoing patients (green dots) at T0 (n=24), T1(n=23), T2 (n=23) and T3(n=10) and in Post TP (orange dots) cohort at T0 (n=3), T1 (n=9), T2 (n=13) and T3 (n=9). (B) Frequency of CD4^+^ T cells reactive to SARS-CoV-2 Nucleoprotein and Membrane protein, defined by expression of CD154 and at least one cytokine among IFN-γ, IL-2 and TNF-α (CD4+CD154+CK+) in TP Ongoing patients (green dots) at T0 (n=8), T1 (n=4), T2 (n=6) and T3 (n=7) and in Post TP (orange dots) cohort at T0 (n=2), T1 (n=5), T2 (n=5) and T3 (n=6). Black lines in A-B represent mean values and grey area delimited by a dotted line in A represents the cut-off value (index=1).

**
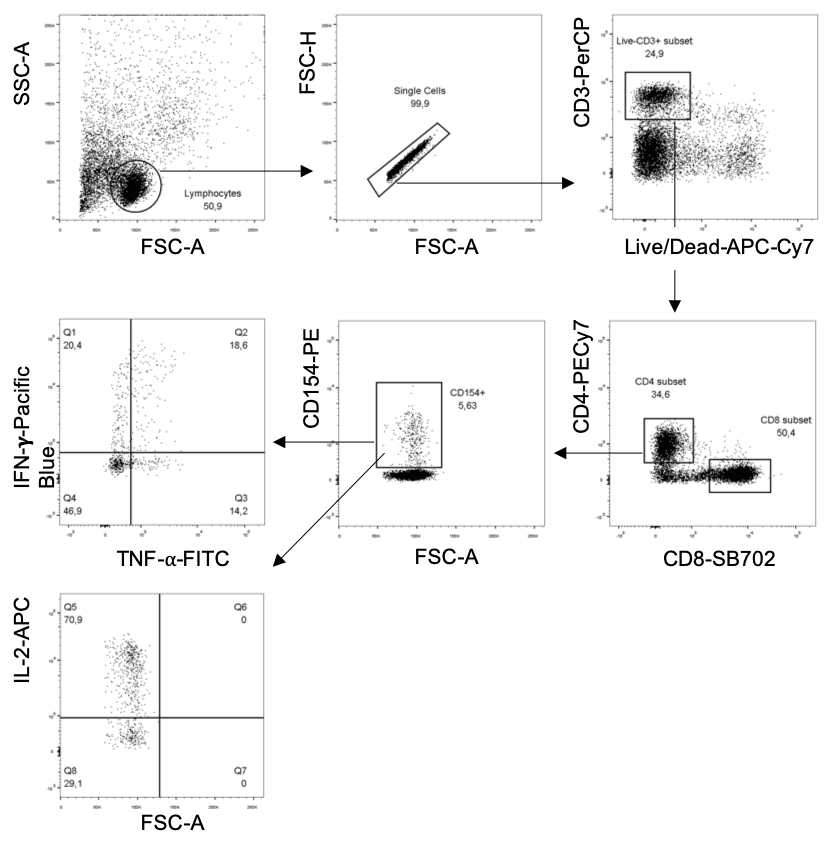
**

**Supplemental Figure 2**. Gating strategy for the identification of SARS-CoV-2-specific T cells.

Lymphocytes were gated based on physical parameters (FSC-A and SSC-A) and doublets were removed using FSC-A and FSC-A parameters. Live T cells were identified by the expression of CD3 and the absence of viability stain (Live/Dead). We then identified CD4+ and CD8+ T cells. CD4+ T cells were evaluated for CD154 expression, and on CD154+ cells we evaluate IFN-γ, TNF-α and IL-2 expression.


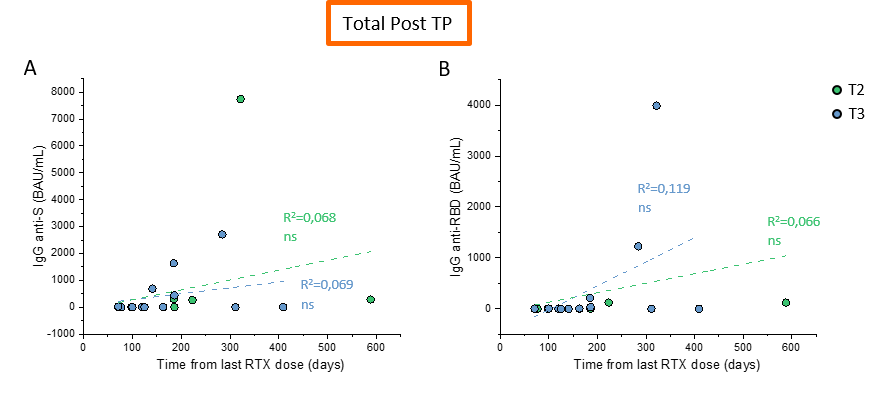
**Supplemental Figure 3**. Correlation between IgG levels and the time from last rituximab dose on post therapy group.

Correlation between IgG anti-S (A) or IgG anti RBD (B) with time from last rituximab dose on Post TP group (n=13) at T2 (1 month after the second vaccine dose; light green dots) or (n=12) at T3 (1 month after the third vaccine dose; light blue dots). Pearson’s correlation coefficients were used to calculate the correlations, no-statistical significance (ns) is reported on each graph.


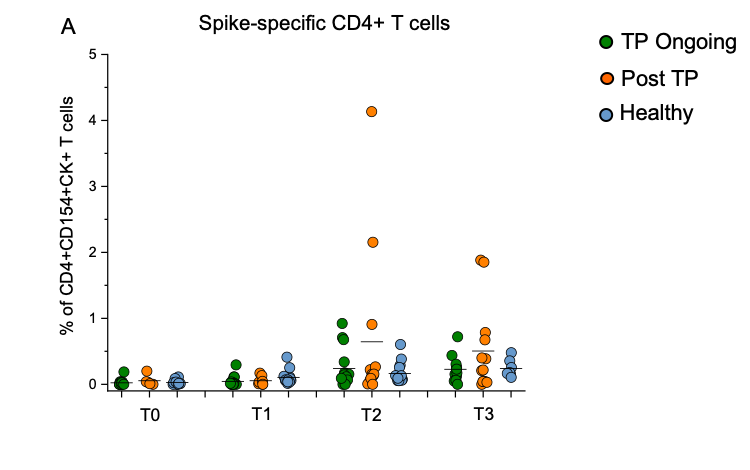


**Supplemental Figure 4.** Evaluation of Spike specific CD4+ T cells after COVID-19 vaccination in Ongoing TP, Post TP patients and Healthy subjects.

(A) Frequency of CD4+ T cells reactive to SARS-CoV-2, defined by expression of CD154 and at least one cytokine among IFN-γ, IL-2 and TNF-α (CD4+CD154+CK+) in TP Ongoing patients (green dots) at T0 (n=14), T1 (n=15), T2 (n=15) and T3(n=10), in Post TP (orange dots) cohort at T0 (n=3), T1 (n=7), T2 (n=13) and T3 (n=12) and in Healthy subjects (light blue dots, n=14) at T0, T1, T2 and T3. The healthy subjects group information were readapted from data showed in Mazzoni et al. JCI 2022.

Mazzoni A, Vanni A, Spinicci M, Lamacchia G, Kiros ST, Rocca A, Capone M, Di Lauria N, Salvati L, Carnasciali A, Mantengoli E, Farahvachi P, Zammarchi L, Lagi F, Colao MG, Liotta F, Cosmi L, Maggi L, Bartoloni A, Rossolini GM, Annunziato F. SARS-CoV-2 infection and vaccination trigger long-lived B and CD4+ T lymphocytes with implications for booster strategies. J Clin Invest. 2022 Mar 15;132(6):e157990. doi: 10.1172/JCI157990.
